# The Role of Connections Between Cellular and Tissue Mechanical Elements and the Importance of Applied Energy in Mechanotransduction in Cancerous Tissue

**DOI:** 10.3390/biom15040457

**Published:** 2025-03-21

**Authors:** Frederick H. Silver

**Affiliations:** Department of Pathology and Laboratory Medicine, Robert Wood Johnson Medical School, Rutgers, The State University of New Jersey, New Brunswick, NJ 08854, USA; silverfr@rutgers.edu

**Keywords:** collagen, epithelial cells, cancer-associated fibroblasts, fibroblasts, macrophages, myofibroblasts, EMT transition, genetic mutations, energy storage, collagen, fibrosis

## Abstract

In the presence of cellular mutations and impaired mechanisms of energy transmission to the attached cells and tissues, excess energy is available to upregulate some of the mechanotransduction pathways that maintain cell and tissue structure and function. The ability to transfer applied energy through integrin-mediated pathways, cell ion channels, cell membrane, cytoskeleton–nucleoskeleton connections, cell junctions, and cell–extracellular matrix attachments provides an equilibrium for energy storage, transmission, and dissipation in tissues. Disruption in energy storage, transmission, or dissipation via genetic mutations blocks mechanical communication between cells and tissues and impairs the mechanical energy equilibrium that exists between cells and tissues. This results in local structural changes through altered regulatory pathways, which produce cell clustering, collagen encapsulation, and an epithelial–mesenchymal transition (EMT), leading to increased cellular motility along newly reorganized collagen fibers (fibrosis). The goal of this review is to postulate how changes in energy transfer between cells and the extracellular matrix may alter local energy equilibrium and mechanotransduction pathways. The changes along with cellular mutations lead to cell and ECM changes reported in cancer, which is postulated to modify mechanical equilibria between cells and their ECM. This leads to uncontrolled cancer cellular proliferation and collagen remodeling.

## 1. Introduction

External and internal forces including gravity and forces involved in locomotion, muscular exertion, and internal tissue tension are essential not only to development but for regulating responses to genetic mutations [1,2,3]. These forces are converted into potential and kinetic energy through deformation of biological macromolecules that make up the cells and the extracellular matrix (ECM) [3,4,5]. At equilibrium, the forces applied to any part of a tissue must be balanced in all directions, and the energy applied to the tissue must either be stored as elastic energy, transmitted away from the site of force application, or dissipated as heat or tissue failure could occur. If energy equilibrium (energy stored = energy released) is not reached, then the tissue will either fail mechanically or the energy can be used to synthesize new tissue components through activation of mechanotransduction pathways [3]. Energy considerations are important to understand how gravitational, internal and external forces, and cellular mutations affect cell and tissue morphological and biochemical changes in cancer [3].

Internal tensile forces provide tissue cohesion, preventing premature mechanical failure, and create a homeostatic equilibrium between tissue synthesis and catabolism [3]. Shear forces applied to cells and tissues result in fluid flow, leading to interstitial fluid rearrangement during mechanical deformation [5]. If fluid flow is blocked, this leads to the build-up of hydrostatic pressure, which is a compressive force. Experiments in which cells are exposed to hydrostatic pressures of 20 kPa or less demonstrate a change in cell metabolism [6].

Transduction of potential and kinetic energy causing changes in cell metabolism in cells and tissues occurs through the activation of a variety of biochemical pathways involved in energy transduction and mechanotransduction. The focus of this paper is to relate how applied potential and kinetic energy resulting from mechanical deformation influences some of the many pathways involved in energy transduction at the cellular and tissue levels in healthy and cancerous tissues.

Forces applied to mammalian tissues are transmitted between cells through cell–cell connections (cell junctions), connections between the cell cytoskeleton and nucleoskeleton, ion channel deformation, intermediate filament connections between cells, and through strong cellular and collagen network attachments found in the ECM. These connections promote a mechanical equilibrium between cells and tissues both locally and in the tissue environment. Mechanical stability of a tissue requires continuous mechanical connections to allow stress transfer without injuring cells and separating cells from their associated ECM. Energy is absorbed, transmitted, and dissipated directly by cells through stretching of (1) attached integrins, (2) cell–cell connections, (3) cell membranes, (4) cytoskeleton–nucleoskeleton connections, and (5) cell–ECM connections. Applied energy leads to activation of genetic material in the cell nucleus via several mechanisms including the Yes-associated protein (YAP) and transcriptional coactivator with PDZ motif (TAZ) [7]. These processes appear to involve hundreds if not thousands of biological molecules and multiple pathways that act together and independently in parallel. Many of the pathways that are affected by mechanical forces have been identified from research studies on vertebrates and invertebrates.

Mechanotransduction is a process by which mechanical energy is converted into biochemical alterations in multiple different pathways. It involves mechanosensors and other molecules that activate signaling pathways, including the mitogen-activated protein kinase (MAPK) family, Hippo, Sonic Hedgehog, PAM, RAS/ROCK, and WNT/beta-catenin (Wingless-related integration site) pathways. Mediators include membrane components such as integrins, beta-catenin, ion channels, cadherins, growth factors, hormone receptors, members of the ROCK/Ras superfamily, and components of the linker of nucleoskeleton and cytoskeleton (LINC) complex. The interrelationship among the different pathways remains to be fully understood; however, all these pathways are influenced by mechanical loading and the energy stored during mechanical deformation.

Integrin-mediated mechanotransduction is one of several ways that external forces are transduced into cellular and tissue changes. It involves direct tensile loading and energy application to the cell membrane via collagen fibril stretching [8]. There is even evidence that mechanical stimulation from surrounding tissues also activates mitochondrial energy generation within the cell [9].

Alterations in the balance of forces and energy applied to cells and tissues are postulated to shift the cell–extracellular matrix mechanical equilibrium and to modify mechanotransduction [3]. The shift in mechanical equilibrium along with cellular mutations can lead to either tissue synthesis, protein structure modifications, or promotion of fibrotic diseases and cancer. The focus of this review is to analyze how applied forces and energy combined with cellular mutations alter energy balances that affect mechanotransduction pathways and the behavior of cancerous tissue.

## 2. Types of Forces Acting on Cells and Tissues That Alter Cell and Tissue Energy Storage, Transmission, and Dissipation

Gravitational and other external forces applied to tissues cause them to undergo deformation that results in energy transfer [3]. This energy is initially stored as molecular deformation; however, it is then transmitted throughout the attached tissues through cellular and ECM connections. In this manner, cells and tissues are always in both local and environmental mechanical communication, and any changes in mechanical stress transfer locally activate mechanotransduction pathways. Ultimately, this energy must be dissipated without causing tissue heating or disruption leading to mechanical failure. Some, if not all, of this applied energy leads to conformational changes in the macromolecules found in cells and tissues and ultimately influences mechanotransduction pathways. For this reason, energy transfer between the external and internal environments complicates the analysis of what is already a complex biomechanical problem.

**Figure 1:**
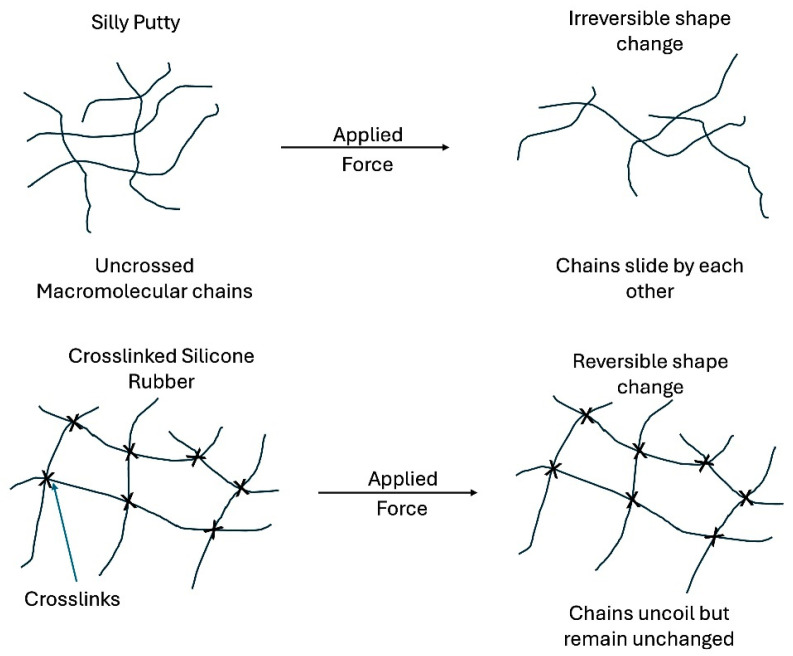
Diagram illustrating how uncrosslinked macromolecular assemblies undergo permanent deformation like silly putty once a force is applied (**top**). Macromolecular chains require chemical crosslinks (xs in diagram) to resist a permanent shape change under applied forces. Once molecules are crosslinked together (**bottom**), the macromolecules can absorb and transmit forces and energy to downstream locations. Applied energy that is stored within crosslinks changes the shape of the tissue in a reversible fashion. For cells and tissues to efficiently store, transmit, and dissipate energy, they need to have mechanical connections that are stable.

The non-ideal mechanical behavior of biological macromolecules and tissue components, including their viscoelasticity, makes interpretation of the problem more difficult. Much literature on mechanotransduction focuses on describing the structure and function of hundreds if not thousands of biological mediators of mechanotransduction; however, little attention is directed to understanding how the dynamic mechanical nature of these molecules involved in mechanotransduction affects biological pathways. While studies on isolated cells provide information on how they respond to mechanical forces, this approach does not consider the intracellular, intercellular, and extracellular connections that are needed for energy transfer and tissue stabilization. Stability of macromolecular mechanical networks in tissues is dependent on strong connections between cells and tissues. These connections are dependent on both the intermolecular chemical and physical crosslinks that transform soft tissues like silly putty (low-molecular-weight silicone chains) into crosslinked silicone networks that can store, transmit, and dissipate energy (see Figure 1). Understanding how energy storage, transmission, and dissipation affect biological pathways involved in mechanotransduction is key to understanding the pathobiology of cancer formation and metastasis. The other factors that complicate interpretation of tissue mechanics, beyond viscoelasticity of the macromolecular components that compose cells and tissues, include anisotropic behavior (properties vary in the x, y, and z directions) and nonlinear behavior (stress and strain are not linearly related).

These biomechanical factors are important, because studies that analyze isolated cells and tissues using classical elastic equations oversimplify the mechanical behavior. Another complicating factor is the flow behavior of biological fluids, whether they be blood or interstitial fluids. The flow behavior and resulting shear and hydrostatic pressure forces generated by biological fluids and dispersions are dependent on the size and shape of the soluble macromolecules and insoluble particles. These factors need to be considered when attempting to analyze viscoelasticity, flow behavior, and connectivity of cells and tissues that are important in regulating mechanotransduction [5]. Some of these mechanical factors will be discussed before attempting to understand how mechanical forces affect mechanotransduction.

## 3. Tensile Forces: How Do They Affect Cells and Tissues?

**Figure 2:**
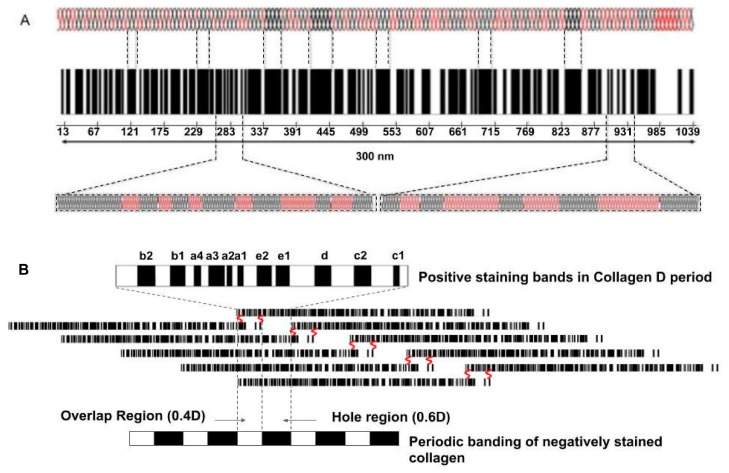
A model depicting how forces and energy applied to collagen molecules are stored by stretching the flexible regions of the molecule that transmits, stores and then can transmit forces and energy through mechanical connections found along the length of a tendon. Collagen molecules are composed of rigid (black) and flexible (red) regions (**A**) and are packed into a quarter-staggered array of five molecules that make up the microfibril (**B**) in the tendon. The numbers under the molecular bar code illustrated in A are the amino acid residue numbers. The flexible and rigid regions shown in A are expanded below the bar code. Initially, under stress, the red regions of the molecule (**A**) are stretched. The banding pattern of the collagen D period is shown in (**B**). The bands a through e represent the polar positively stained charged amino acid residue bands characteristic of collagen fibrils that are stretched after a mechanical force is applied that arises from alignment of all the flexible regions in the collagen molecules. The negatively stained bands, showing the hole and overlap regions at the bottom of (**B**), illustrate locations where stain penetration occurs into the hole region of the five-membered microfibril devoid of amino acids in negatively stained collagen fibrils [10]. Energy is stored by stretching the positively stained regions of the fibril that reversibly stretches the hole region. Since fibroblasts sit on collagen fibers on the tendon surface, they are sheared when the fibers are stretched as the fibers slide by each other (Figure 3). The covalent crosslinks that connect all collagen molecules into a mechanical network in tendons are shown in red.

The effects of tensile forces on cells and tissues can be considered by analyzing how muscular forces applied to tendons stretch the aligned collagen fibers and result in applying shear forces to the attached fibroblasts. When tension is applied to the ends of a tendon, the tendon length increases by stretching the collagen fibrils and fibers, which in turn causes molecular deformation of collagen triple helices, leading to energy storage [10]. This leads to conformational changes, molecular deformation, and sliding of the collagen molecules, fibrils, and fibers by each other (see Figure 2) that is reversible [11].

**Figure 3:**
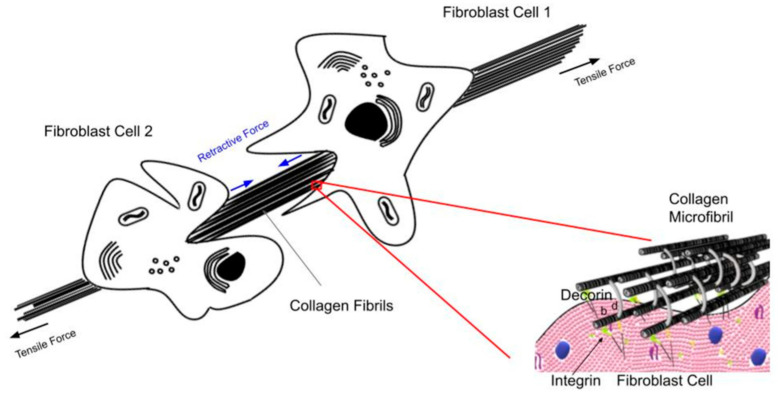
Diagram illustrating the tensile forces acting on collagen fibers and cells. Collagen fibers are stretched in tension under normal physiological conditions. This causes the fibroblasts attached to the collagen fibers to generate a retractive cellular shear force via attachment through integrin and decorin molecules. These attachments serve as a feedback mechanism to reflect the current level of tensile loading in the tissue. Increased external loading stretches the collagen fibers, increasing their internal energy as well as increasing the energy stored in the attached cells, upregulating mechanotransduction under normal physiological conditions. This explains why weightlifting increases muscle size and generates more skin to cover the larger muscles. Note the location of integrin–collagen–fibroblast attachments as well as collagen–decorin attachments at the d bands of collagen fibrils [3,5].

The application of forces to the cell membrane during collagen fiber stretching causes stretching of cytoplasmic components as well as the cell cytoskeleton and nucleoskeleton through their mechanical connections [12]. Mechanical energy applied in this manner is further transferred between cells through stretching of cell–cell junctions and ion channels. Any defects in cell junctions alter local mechanotransduction processes. Stretching of the collagen fibers and their constituent fibrils causes energy to be transferred through integrins to the interior of the cell [3,5]. Molecular stretching of collagen molecules also leads to rearrangement of loosely bound water molecules that surround the collagen fibers. This results in fluid movement (flow) and the application of shear and compressive forces to cells and the surrounding ECM. The flow behavior of the fluid in interstitial spaces depends on the molecular structure of the molecules in the fluid.

The mechanical behavior of large macromolecules that make up cells and tissues depends on the rate of force application, the degree of macromolecular stretch (strain), and the time after loading and unloading [13]. Beyond these influences, the viscosity and flow behavior of the interstitial fluids and blood are dependent on the cell and macromolecular concentration and the shape factors of the soluble and insoluble elements in the fluid [5]. Therefore, the interpretation of the effects of mechanical loading on cell and tissue behavior becomes somewhat more complicated.

## 4. How Do Cellular and Macromolecular Size and Shape Affect Interstitial Fluid Behavior as Well as Tissue Stiffness?

The size and shape of the constituent macromolecules influence mechanotransduction by altering tissue stiffness, fluid viscosity, cell motility, and flow behavior. The relationship between macromolecular structure and stiffness is related to the conformation of individual macromolecular chains and the hydrogen bonding patterns within a chain [14]. Macromolecules with a large axial rise per residue have higher stiffnesses compared to more random chains due to the hydrogen bonding within or between chains [3,5,14].

The ability of cells and extracellular matrix to deform under shear, tensile, and compressive (including hydrostatic pressure) loading is complicated by how cells and tissues are attached to each other. The relationship between cell motility and shape suggests that cell shape plays a role in defining effective tissue viscosity [14]. Interstitial fluid viscosity in tissues is dependent on the shape factor of constituent cells and macromolecules, which influences how quickly fluid will flow and how much pressure is generated when an external force is applied. In this manner, highly viscous fluid flow will alter shear forces exerted on neighboring cells and tissues ultimately affecting mechanotransduction. Stiff molecular chains such as that exhibited by fibrillar collagen increase the interstitial fluid viscosity compared to flexible chains that are found in molecules like albumin [14,15,16]. Macromolecular size and shape influence tissue viscosity and contribute to hydrostatic pressure build-up when flow is compromised, such as when vessels are blocked. Mutations that cause increased mucus viscosity in cystic fibrosis result in defective airway clearance due to increased fluid viscosity [17].

Fluid flow in bone and the cardiovascular system influence tissue remodeling, cellular migration, and mechanotransduction [18,19]. Changes in red blood cell shape in sickle cell anemia impede blood flow and alter oxygen distribution to peripheral tissues. Decreased red blood cell deformability and aggregation have been shown to correlate with increased hemolysis [20].

While cell and macromolecular shape are important aspects that determine biomechanical behavior, the way cells and tissues are connected is also an important aspect of energy storage and mechanical continuity required for tissue mechanical homeostasis. Both tight and adherence cell junctions are important for the mechanical properties of confluent endothelial sheets [20]. Beyond cell–cell mechanical connections [21], covalent crosslinking of collagen fibers in the ECM is needed to transmit mechanical energy between cells and tissues [22]. Energy transfer between cells and the ECM therefore requires the formation of a continuous network of connections. The absence of these connections in the dermatosparaxis Ehlers Danlos syndrome results in skin fragility, tearing, and laxity [23].

Energy storage and transmission between cells and the ECM are important for establishing a mechanical equilibrium (forces in all three directions cancel each other out) in tissue without causing cellular disruption and tissue alterations. It is interesting to note that human cancers do not tear mechanically when they stiffen because of collagen fibrosis and their inability to dissipate the stored energy. This suggests that fibrosis is a result of disturbance of the mechanical equilibrium via altered mechanotransduction, leading to the inability of cancerous tissues to transfer energy to the surrounding tissues.

## 5. Cellular Molecules and Assemblies Involved in Mechanotransduction

There are many cellular and tissue molecules that influence mechanotransduction (Table 1). The interrelationships between the different molecules and pathways are not currently totally clear. The cell cytoskeleton is made up of many different macromolecules and assemblies including actin filaments (microfilaments), intermediate filaments (keratin), and microtubules (tubulin). Energy applied to the cell membrane deforms these cell structures and modifies their behavior. This occurs through energy transmission via stretching intracellular and intercellular connections. Energy application to cells causes activation of mitochondria, actomyosin–myosin interactions, and actin polymerization influencing cell spreading and elongation [24]. Applied mechanical energy alters cellular processes (Table 2). This energy is then available to activate mechanosensing transcription factors YAP/TAZ, MAPK, and HIPPO pathways discussed below.

Actomyosin-generated tension at cell adherens junctions is required for cell-density-dependent inhibition of proliferation of normal skin keratinocytes. This tension is lost after an epithelial–mesenchymal transition (EMT) occurs in cancerous lesions. Cell junctions that link cells together support cell proliferation in normal tissue, while defects in these junctions disrupt homeostasis and are commonly found in genetic mutations leading to cancer [25].

Actomyosin contractility is also required for cancer cell migration and invasion; it is activated by ROCK signaling and by other signaling pathways that are crucial for physical remodeling of the ECM favoring tumor dissemination. Matrix stiffening via TGF-beta and YAP activation in cancer-associated fibroblasts (CAFs) promote ECM remodeling [26].

Alteration in keratin in intermediate filaments connected to the cell cytoskeleton is reported to drive melanoma cell motility and that in other cancer cells. Keratin tumor markers (K8, K18, K19) are found in peripheral blood [27,28]. Energy stored and transmitted between cells through intermediate filament connections may help transfer mechanical energy between cells and lead to equilibrium.

Changes in intracellular and intercellular forces alone do not explain alterations in cellular behavior observed in cancer. Mutations of tumor suppressor TP53 protein are found in many forms of human cancer and are associated with increased tumor cell invasion and metastasis [29]. In cancer cells, impairing p53 can promote increased myosin X expression, causing cell adhesion inhibition and tumor progression [29,30].

## 6. Cells Involved in Mechanotransduction

A variety of cells are involved in changes observed in mechanotransduction (Table 3). Epithelial cells act as sensors for mechanical forces through adherens and tight junctions which connect neighboring cell cytoskeletons. They trigger changes in cancer cell behavior, such as gene expression, migration, and barrier function [31]. In concert with fibroblasts, their changes influence alteration in the ECM surrounding cancer cells. Fibroblasts can transition into myofibroblasts under the influence of factors released by several pathways involved in mechanotransduction. Release of TGF-beta 1 contributes to ECM synthesis and matrix rigidity [3]. Mutations in melanocytes, basal cells, and squamous cells found in the skin in concert with cancer-associated fibroblasts and immune cells lead to skin cancer progression.

Tumor-associated macrophages are found in the tumor microenvironment and promote angiogenesis, ECM remodeling, cancer cell proliferation, metastasis, and immunosuppression [3]. Cancer-associated fibroblasts (CAFs) influence tumor behavior by secreting molecules that cause cellular proliferation, degradation of the ECM, and release of extracellular vesicles, and alter ECM architecture after direct contact with cancer cells [32]. CAFs and tumor-associated macrophages together exert protumor functions [33]. During interactions between CAFs and the ECM, CAFs apply traction forces deforming the ECM, leading ECM remodeling and stiffening [34,35,36,37,38,39]. Increased traction forces generated by CAFs and other cells in the ECM alter the force and energy balances, affecting cell–cell and cell–ECM communication via the activation of mechanotransduction pathways. The loss of these connections and subsequent alterations in force and energy balances appear important in understanding cancerous tissue behavior.

Gene mutations alter the behavior of fibroblasts, CAFs, and other cells in the ECM. The number of driver events that cause gene mutations required for tumor initiation and progression varies. This may reflect the redundancy and co-stimulation of several mechanotransduction pathways required for tumor growth. Only three sequential mutations are required to develop lung and colon adenocarcinomas [40]. How these mutations along with activation of CAFs and macrophages result in mechanical decoupling between cells and the ECM is unclear. However, below, we see that several pathways can alter mechanotransduction and affect the outcome of tissue responses after mechanical force application. Perhaps several mutations are required to alter enough of the mechanotransduction pathways to shift cellular and ECM behavior to that of cancerous tissue.

## 7. Cellular Junctions Involved in Mechanotransduction

The ability of cells including epithelia to support loads and energy applied externally to other cells requires significant cell stiffness beyond that exhibited by single cells. Single cells can be pipetted without rupturing, while cellular sheets with cell–cell connections are more stable to mechanical loading and can store additional mechanical energy without flowing or tearing. However, even cellular sheets require adhesion to the ECM to result in optimum mechanical stability. Cellular junctions are very important in providing stress and energy transfer continuously in cell–cell connections and between cells and the ECM to maintain a mechanical equilibrium. Changes in cell junctions may precede changes that are observed in cancer (Table 4).

Normal epithelial cells maintain tissue structure by adhering to each other and the ECM. Adherence is mediated through cell junctions via adherens junctions, tight junctions, anchoring junctions, gap junctions, and demosomes. Breakdown of adherens junctions, tight junctions, and desmosomes facilitate metastasis of squamous cell carcinoma [41]. Disruption of tight junction proteins and their barrier function can lead to cancer metastasis of other cells [42,43], suggesting that constant mechanical communication between cells through normal junctions is needed to maintain local mechanical and energy balances.

Anchorage-dependent cells, such as epithelial cells, cannot survive or divide unless attached to a surface. In contrast, cancer cells become anchorage-independent. A variety of signaling pathways are upregulated during cell detachment in cancerous tissues that are required for anchorage-independent survival [44]. Molecules involved in cell junctions that become very important in maintaining cell-to-cell attachments include cadherins, integrins, selectins, and immunoglobulins. Disruption of cell–cell adhesion molecules between melanocytes and keratinocytes is reported in melanoma cells [45]. Cancer cells may create new local force and energy balances, leading to cell clustering and fibrosis.

Adherens junctions are coupled to the cytoskeleton through cadherin complexes. They are made up of cadherins and catenins and are essential to maintain epithelia cell homeostasis. They are downregulated in many cancers, promoting tumor progression [46]. In contrast, overexpression of cell junctions also induces malignant transformation and protects epithelial cells from anoikis (programmed cell death after detachment from the ECM). Cadherins are major cell–cell adhesion molecules in tumors. When they are disrupted or altered, disaggregation of cells promotes invasion and metastasis of cells [47].

Desmosomes are cell–cell connections that connect intermediate filaments and the cytoskeletons of neighboring cells. They respond to external mechanical stress [48,49]. Unlike adherens junctions and tight junctions, desmosomes are coupled with intermediate filaments. They are involved in intercellular adhesions of epithelial cells [49]. Downregulation of desmosome proteins in various cancers promotes tumor progression [50,51], perhaps by decoupling mechanical connections between cells.

Tight junctions (TJs) play a role in adhesion of cells to each other [52]. Changes in expression of or distribution of TJ proteins can lead to cancer cell invasiveness and spreading. Communication and crosstalk in the tumor microenvironment are maintained by tight junctions. Low expression of TJs is observed among highly metastatic cancers.

## 8. Cellular Structures Involved in Mechanotransduction

Cell membrane structures associated with mechanotransduction include adherens junctions, hormone and growth factor receptors, and cell membrane channels. Focal adhesions are large protein complexes that connect the cell cytoskeleton to the ECM through integrins. Focal adhesions are made up of groups of aggregated collagen–integrin complexes. In cancer, changes in many of the cell adhesion molecules are involved in the cell–ECM linkage.

Hormone and growth factor receptors also play a role in mechanotransduction. Hormone receptors on breast cells pick up estrogen or progesterone signals that promote growth. These receptors are activated in cancer when cells produce proteins that bind to hormone receptors and upregulate mechanotransduction [53]. Cellular components involved in mechanotransduction are listed in Table 5.

Piezo, TREK, transient receptor potential (TRP), and big potassium (BK) channel families also contribute to mechanotransduction. Piezo channels are 10× more sensitive to membrane tension when tethered to the extracellular matrix. Piezo channels are bound to the actin cytoskeleton by the E-cadherin complex, and the absence of E-cadherin or beta-catenin deactivates Piezo1 channels. Mechanical compression activates Piezo1 channels in enhanced breast cancer cell invasion, which leads to both cellular events and matrix degradation. Piezo1 has an influence on the epithelial–mesenchymal cell transformation (EMT) and is one of the causes of cancer dissemination [54,55].

TRP channels are involved in cell proliferation, migration, invasion, and angiogenesis related to cancer progression [56]. TRP-4 channels are force-sensing; transduction may be mediated by the interactions of the TRPP1/TRPP2 complex with the extracellular matrix, focal adhesions, the cytoskeleton, or other mechanosensitive channels like Piezo1 [56,57].

Polycystins represent a family of proteins that belong to the machinery of the mechanotransduction process [58]. Polycystins are expressed in a variety of epithelial cells and regulate calcium flux. They are found in epithelial cell membranes in the liver, pancreas, kidneys, and breast. Polycystins (polycystin-1, PC1; polycystin-2, PC2) respond to changes of extracellular mechanical cues, and mediate pathogenic mechanotransduction and cyst formation in kidney cells.

## 9. Signaling Pathways Involved in Mechanotransduction

There are numerous signaling pathways influencing mechanotransduction that are affected by cancer. Because of the large number of pathways and the extensive literature available, only some of these pathways will be discussed here. Below, we discuss MAPK, Hippo, JUNC, p38, ERK 5, RAS/ROCK, PAM, HIPPO, Sonic Hedgehog, and WNT pathways (see Table 6). Only a summary of the different pathways is discussed in this section, as the exact mechanisms by which each type of mechanical force affects mechanotransduction pathways are complicated by crosstalk between the different pathways.

Mitogen-activated protein kinase (MAPK) signaling pathways are key mediators of eukaryotic transcriptional responses to extracellular signals. The four major MAPK pathways in mammalian cells include ERK/EGF/ERK1/2 (extracellular signal-regulated kinase), cJUN N-terminal kinase (JNK), p38, and ERK 5 [59]. Integrins couple to the cytoskeleton through F-actin binding proteins, such as talin and vinculin. MAPK pathways can be activated by mechanical forces applied to the cell surface through integrin–collagen interactions applied through focal adhesions. Integrin–collagen interactions activate the MAPK/EGF/ERK1/2 pathway. Abnormal regulation of integrin receptors allows cancer cells to break down tissue borders [59].

Integrins are cell surface adhesion receptors that exhibit various cellular functions crucial to initiation, progression, and metastasis of solid tumors [60]. Integrins associate with different intracellular signaling pathways, including the small MAPK, GTPases, RhoA and Rac, the Hippo signaling pathway, and focal adhesion kinase (FAK) and SRC (a protein kinase that plays a role in development and progression of several cancers).

One of the MAPK pathways is ERK (ERK1/2, ERK, or EGF pathway), a central regulatory pathway that plays a key role in cancer development and progression. Its activity is deregulated in many cancers [61]. JNKs are protein kinases that regulate many biological processes including inflammation, morphogenesis, cell proliferation, differentiation, survival, and cell death. Persistent activation of JNKs is involved in cancer development and progression [62]. P38, Rho/Rock/RAS, and Smad2 and Smad3 are required for transforming growth factor (TGF) tissue growth inhibition [63]. Rho kinase (ROCK/RAS) blockage increases cancer phagocytosis and induces antitumor immunity, leading to suppression of tumor growth in models. Rho-associated kinases (ROCKs are downstream effectors of RhoA) have been implicated in tumor mobility, invasion, and growth [64].

The Ras gene family encodes proteins that play a role in cell signaling and are frequently mutated in cancer [65]. Studies reveal that Ras malignant transformation protects epithelial cells from anoikis (cell death), while the Ras/ERK pathway inhibitors reverse anoikis resistance. When RAS genes are mutated, cells grow uncontrollably and evade cell death [65,66,67].

Extracellular signal-regulated kinase 5 (ERK 5) belongs to the mitogen-activated protein kinase (MAPK) family expressed by eukaryotic cells involved in cell survival, proliferation, migration, and differentiation. ERK 5 has been implicated in the onset and progression of cancers [68]. During tumor progression, ERK5 signaling enhances angiogenesis to allow the supply of oxygen and nutrients to cancer cells and facilitates their spreading [68].

The P13K/AKT(PAM) signaling pathway is a highly conserved signal transduction network in eukaryotic cells that promotes cell survival, cell growth, and cell cycle progression. The two major proteins in this pathway are P13K and AKT. This pathway is the most frequent pathway activated in human cancer [69]. Alteration in growth factor receptor family genes leads to activation of PAM genes and cancer treatment failure [69].

The Hippo pathway is a highly conserved signaling pathway that controls organ size, tissue homeostasis, and cancer development [70]. The Hippo signaling pathway restricts cell proliferation in animal tissues by inhibiting Yes-associated protein (YAP or YAP1) and TAZ. YAP/TAZ are part of the Hippo pathway and are transcriptional regulators activated in human malignancies. They are important for cancer initiation and growth of most solid tumors [71,72].

The Sonic Hedgehog signaling pathway (SHH or HH) plays a role in embryogenesis and tissue homeostasis. Aberrant activation of this pathway has been linked to different diseases including cancer. Activation of the pathway through mutations is associated with development and progression of several cancers [73].

WNT/beta-catenin signaling promotes differentiation of many cancer stem cells, which can be precursors of mature cancer cells [74]. The WNT/beta-catenin pathway is a highly conserved pathway that regulates cell proliferation, migration, genetic stability, apoptosis, and tissue homeostasis. It is also involved in diseases including cancer [74,75].

A recent review suggests that the PAM axis is the most frequently activated signaling pathway in human cancer [76], and crosstalk between the PAM, MAPK, and WNT/beta-catenin pathways has been reported to be responsible for controlling critical functions in human cancer cells [77].

## 10. Molecules Involved in Integrin-Mediated Mechanotransduction Include FAK, Talin, and Vinculin

A variety of molecules have been implicated in integrin-mediated mechanotransduction, including focal adhesion kinase (FAK). FAK recruitment and activation in integrin adhesions constitute one of the early stages in the cellular response to external mechanical stimuli. FAK and/or Src are highly expressed and/or activated in many cancers and lead to nuclear activation through YAP/TAZ. Hyperactivation of FAK/Src signaling can help cancer cells promote cell survival from signals derived from ECM-integrin adhesions. Src is a non-receptor tyrosine kinase that is deregulated in many cancers [78,79]. Talin is a component that plays a role in integrin-mediated signaling. Talin-1 mutations can affect cell behavior and may also contribute to cancer progression [80]. Vinculin is a component of both focal adhesions and adherens junctions. It binds to talin and alpha- and beta-catenin, among other binding partners. Vinculin is a key to adhesion related to cell–ECM and cell–cell junctions. The role of vinculin in cell attachment and detachment during migration is critical, indicating that the adherens junction cell–ECM adhesion can favor or inhibit the metastatic cascade [81,82].

Kindlins are mechanosensitive molecules transducing extracellular mechanical cues to biochemical signals. Cancer-specific co-alterations between kindlins and proteomic components involved in mechanotransduction have been observed in advanced tumor stages through activation of cancer-related pathways [82,83]. Alterations in endothelial–mesenchymal transition (EMT) markers are associated with kindlin activity.

## 11. Role of Nuclear Membrane in Mechanotransduction

Cytoskeletal forces in actin filaments are transferred across the nuclear envelope to the nuclear lamina through Sun and KASH proteins that make up the LINC complex [84]. An intact LINC complex is required for nuclear positioning, cell polarization, and normal propagation of cytoskeletal forces [85]. The LINC complex is a series of proteins that physically link the nucleoskeleton to the cytoskeleton. It allows force transmission from the cytoplasm to the nucleus that affects nuclear spacing and genetic regulation [86]. It comprises Sad-1 and UNC-1, SYNE homology domain proteins providing a physical coupling between the cytoskeleton and nucleoskeleton [87].

The linker of nucleoskeleton and cytoskeleton complex (LINC) is involved in cancer progression, as it regulates the transmission of forces between the cytoskeleton and nucleoskeleton [88]. Aberrations in the structure and expression of nuclear envelope proteins have been linked to tumor growth and migration [89].

## 12. Role of Epithelial–Mesenchymal Transition (EMT) and Extracellular Matrix (ECM) in Mechanotransduction

The epithelial–mesenchymal transition (EMT) is a biological process that allows an attachment-dependent epithelial cell to undergo biochemical changes to assume a mesenchymal phenotype that does not require attachment to the ECM for survival [90]. EMT leads to new cellular properties that include enhanced cell migration, invasiveness, resistance to apoptosis, and increased production of ECM components [91]. EMT allows solid tumors to become more malignant, increasing their invasiveness and metastatic activity [92]. The SRC family of kinases are key players in tumor progression. They play a supporting role of EMT in invasion and metastasis [93]. One way by which ECM stiffness impacts EMT is via modulating the response to TGF-beta 1, a key biochemical inducer of EMT. At high stiffness, TGF-beta 1 is found to promote EMT, while at low stiffness, it leads to apoptosis [94]. TGF-beta 1 promotes both fibroblast proliferation and collagen synthesis by fibroblasts in keloids [94]. It contributes to differentiation of T cells and to deposition of tumor stroma and collagen production and fibrosis [95].

Dense aligned collagen type I fibers (fibrosis) are associated with cancer invasion led by protrusive tumor cells. The ECM undergoes dramatic remodeling during tumor growth and is degraded and replaced with an ECM with a higher-density collagen (fibrosis) with increased stiffness [96]. Collagen fibrils and fibers interact with integrins through integrin-mediated pathways to upregulate mechanotransduction and ECM stiffening. In rare round desmoplastic aggressive tumors, CAF production of type I collagen and other ECM components leads to increased tumor stiffness. In these cancers, collagen fibers are oriented parallel to the surface of the tumor [97]. In invasive tumors, the collagen fibers are oriented perpendicular to the tumor border and in the general direction of cellular invasion. Liver fibrosis has been related to changes in tissue viscoelasticity and has been used to follow cirrhosis and liver cancer [98].

## 13. Summary of the Effects of Mechanical Forces and Energy Storage on Cell–Cell and Cell–Matrix Interactions on Mechanotransduction

**Figure 4:**
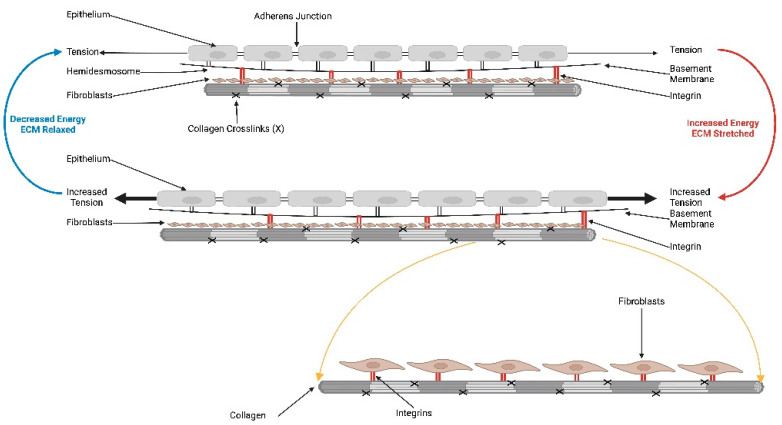
Diagram postulating the role of mechanical force and energy equilibria that drive cell and tissue tensional homeostasis. At equilibrium, all the forces acting on a tissue must sum to zero, and the energy must be either stored, transmitted away from the area of force application, dissipated via release of heat, or lead to upregulation of mechanotransduction. Unless this happens, energy is dissipated by tissue tearing. When normal external and internal forces work upon cells and tissues, the energy is transmitted between epithelial cells, from epithelial cells to the basement membrane, from the basement membrane to collagen fibrils, from collagen fibrils to fibroblasts, and between collagen fibril connections (**Top**). Energy applied to epithelial cells and the ECM is transmitted throughout local and surrounding tissues by stable mechanical connections, leading to force and energy balances. These equilibria dictate the level of activation of mechanotransduction pathways leading to any cellular and tissue responses. In this manner, locally applied forces and energy affect local cells and tissues as well as cells and tissues at a distance away from the site of force application due to the continuous mechanical connections (**Bottom**). When the local force and energy application increases, this upregulates mechanotransduction pathways, generating local cellular proliferation and ECM synthesis, as well as energy dissipation through cellular and ECM connections away from the local site. When cancer mutations occur, this leads to breakage of cell–cell and/or cell–ECM connections. This impairs local energy dissipation, leading to upregulation of mechanotransduction pathways and excessive cellular proliferation of CAFs, macrophages that activate tumor EMT under the influence of mutant cells. This leads to modification of the tumor microenvironment due to the loss of continuous connections between cells and the ECM under the influence of local cellular mutations.

The role of biochemical factors affecting mechanotransduction pathways has been documented in detail in the literature. In contrast, little attention has been focused on the role of energy storage, transmission, and dissipation in cells and tissues. The presence of gravitational external forces and internal cellular and tissue forces influences cell and tissue behavior and appears to be part of a local and environmental energy balance cycle (see Figure 4). This cycle provides a mechanism to respond to changes in forces and energy applied to tissues. While both external and internal forces and energy are transduced into tissue deformation and macromolecular conformational changes in cells and tissues, little is understood of how these changes affect the mechanotransduction pathways that occur in cancer. Macromolecular component conformational changes can explain how some of the applied energy is stored and mechanotransduction is activated. Some of the stored energy must then be transferred to the attached cellular and tissue components through cell junctions and the nucleoskeleton. This energy transfer between cells and from cells to the ECM results in a mechanical energy equilibrium that can be altered locally and transmitted to neighboring cells and tissues. In the presence of cellular genetic mutations that alter cell junction, cell membrane composition, and connections between neighboring cells and tissues, these mechanotransduction pathways are altered, resulting in locally uncontrolled proliferation and migration of cancer cells. In this case, energy transfer shifts from an overall tissue equilibrium to a local equilibrium regulated via mutated cells and cancer-associated fibroblasts. This local energy shift upregulates several of the different mechanotransduction pathways to locally alter the structure and function of the affected tissue. This cuts off the connections for the mechanical energy equilibrium of neighboring tissues. This isolates the local tumor environment, including the mutated cancer cells, CAFs, myofibroblasts, and ECM, leading to cell clusters and local fibrosis.

Figure 4 shows a proposed model of the force and energy equilibrium cycle that regulates local and attached tissue metabolism. The force and energy equilibria between cells and the ECM are driven through cell–cell and cell–ECM attachments and cycles constantly in response to applied forces. This allows local force and energy changes to respond by changing tissue structure and function. In the presence of cellular mutations, the local force and energy balance is decoupled from the environmental balance, leading to local structure and function changes through alterations in mechanotransduction, which allow for uncontrolled cellular reproduction and migration.

## 14. Conclusions

In the presence of cellular mutations and impaired mechanisms of energy transmission to the attached cells and tissues, excess energy is available to upregulate some of the mechanotransduction pathways that maintain cell and tissue structure and function. The ability to transfer applied energy through integrin-mediated pathways, cell ion channels, cell membrane, cytoskeleton–nucleoskeleton connections, cell junctions, and cell–extracellular matrix attachments provides an equilibrium for energy storage, transmission, and dissipation in tissues. Disruption in energy storage, transmission, or dissipation via genetic mutations blocks mechanical communication between cells and tissues and impairs the mechanical energy equilibrium that exists between cells and tissues. This results in local structural changes through altered regulatory pathways, which produce cell clustering and collagen encapsulation and an epithelial–mesenchymal transition (EMT), leading to increased cellular motility along newly reorganized collagen fibers (fibrosis). A model is proposed that describes the role of mechanical forces and energy storage and transmission that drive cell and tissue tensional homeostasis.

## Figures and Tables

**Table 1:** Molecules involved in cellular and tissue mechanotransduction.

Structural Component	Effectors of Mechanotransduction
Cell membrane	Integrins, ion channels, growth factor receptors, cadherins, catenins
Cell cytoskeleton	Actin, myosin, intermediate filaments, microtubules
Cell junctions	Focal adhesions, tight junctions, gap junctions, adherens junctions, desmosomes
Nucleoskeleton	LINC proteins

**Table 2:** Outcome of force and energy application to cellular components.

Component	Effect
Actin filaments	Change in actin–myosin interactions, actin polymerization, cell spreading, actomyosin tension at cell junctions, cancer cell migration
Mitochondria	Activation of mitochondria and energy production
Intermediate filaments	Increased cell motility of cancer cells
Microtubules	Increased pulling forces between cells

**Table 3:** Cells involved in mechanotransduction.

Cell	Role in Mechanotransduction
Cancer-associated fibroblasts (CAF)	Cause cellular proliferation, degrade ECM, applyand generate traction forces, deform ECM
Epithelial	Act as mechanosensory, trigger changes in cell behavior
Fibroblast	With epithelium, alter cell and tissue organizationTransition into myofibroblasts
Melanocytes, basal, and squamous cells	Mutations cause uncontrolled growth
Myofibroblast	Applies tension to ECM, contributes to ECM synthesis and stiffness
Macrophage	Promotes angiogenesis, ECM remodeling, cancer cell proliferation, metastasis, immune suppression

**Table 4:** Role of cellular junctions in cancer.

Junction	Role in Cancer
Adherens	Initiate cell–cell contacts through cadherins and the cytoskeleton; mutations facilitate metastasis
Desmosomes	Connect cells through intermediate filaments, downregulated in cancer
Gap	Allow ion and small-molecule cell–cell communication, lost or reduced in cancer
Tight	Watertight junction, loss in cancer leading to metastasis

**Table 5:** Mechanisms by which cellular structures influence mechanotransduction.

Structure	Role in Mechanotransduction
Cell membrane and associated components	Focal adhesions provide connection to ECM, growth factor and hormone receptors and membrane channels activate pathways
Cell junctions	Provide connections between cells and activate mechanotransduction pathways
Anchoring junctions	Activate integrin dependent mechanotransduction
Actin microfilaments, intermediate filaments, microtubules	Support the cytoskeleton and form cellular connections
Hormone and growth factors	Stretching and mutations activate mechanotransduction
Ion channels	Respond to membrane tension, influence EMT and remodeling

**Table 6:** Signaling pathways involved in mechanotransduction.

Pathway	Role in Mechanotransduction
MAPK	ERK (EGF/ERK ½), cJun, p 38, ERK 5 involved in gene transcription, cell differentiation, cytokine release, and apoptosis
Hippo	Limits cell proliferation, inhibits YAP/TAZ activity, YAP/TAZ activated in cancer
PAM (p13/AKT)	Activated in human cancers, influenced by growth factor pathways
RAS (ROCK)	Encode proteins involved in cell signaling and mutation, cause uncontrolled growth and invasion and cell death
Sonic Hedgehog	Activated in development and progression of several cancers
WNT/beta-catenin	Promotes differentiations of cancer stem cells that are precursors of mature cancer cells

## Data Availability

Not applicable. The information used in this review can be found in the references cited.
